# Structural and Functional Brain Alterations Induced by Noise Exposure: A Comprehensive Review

**DOI:** 10.3390/neurosci7040075

**Published:** 2026-06-24

**Authors:** Hanna Valeria Venegas-Mora, Octavio Ispanixtlahuatl-Meraz, Diana Emilia Martínez-Fernández, Irene Guadalupe Aguilar-García, David Fernández-Quezada

**Affiliations:** 1 Instituto de Neurociencias Traslacionales, Departamento de Neurociencias, Centro Universitario de Ciencias de la Salud (CUCS), Universidad de Guadalajara (UdeG), Sierra Mojada 950, Guadalajara 44340, Mexico; 2 Laboratorio de Carcinogénesis y Toxicología, Unidad de Biomedicina, Facultad de Estudios Superiores Iztacala, Universidad Nacional Autónoma de México, Av. de los Barrios No. 1, Los Reyes Iztacala, Tlalnepantla de Baz, Estado de México 54090, Mexico; 3 Instituto Transdisciplinar de Investigación y Servicios (ITRANS), Universidad de Guadalajara (UdeG), Zapopan 45150, Mexico

**Keywords:** noise exposure, cognition, behavior, brain, neuroplasticity

## Abstract

Noise exposure has become an increasingly prevalent public health concern, with effects extending beyond the auditory system. Accumulating evidence indicates that chronic noise exposure induces both structural and functional alterations in the central nervous system, ultimately affecting cognitive and emotional processes. This review summarizes the impact of noise on key brain regions, including the hippocampus, prefrontal cortex, and auditory cortex. Structurally, noise exposure is associated with reduced neurogenesis, dendritic remodeling, synaptic loss, alterations in white matter and changes in glial activity. Functionally, it disrupts synaptic plasticity mechanisms—such as long-term potentiation and long-term depression—as well as neuronal connectivity, leading to impairments in higher-order cognitive and behavioral functions. These effects are mediated by interconnected mechanisms, including activation of the hypothalamic–pituitary–adrenal axis, neuroinflammation, oxidative stress, and alterations in neurotrophic signaling.

## 1. Introduction

Noise has become increasingly prevalent in modern environments and is now recognized as a significant stressor with important implications for human health. Although it has traditionally been associated with alterations in the auditory system, accumulating evidence indicates that noise exposure also produces non-auditory effects on the central nervous system (CNS), affecting brain structure, function, and behavior. These effects are particularly relevant under chronic exposure conditions, in which noise acts as a persistent stressor capable of inducing both short- and long-term neurobiological changes [1].

The impact of noise on the brain involves both direct and indirect mechanisms. Acoustic information is processed through the auditory system and distributed across interconnected cortical and subcortical networks, enabling noise to influence regions beyond classical auditory pathways, including the hippocampus, prefrontal cortex, thalamus, and other limbic structures [2]. In parallel, noise exposure activates stress-response pathways, particularly the hypothalamic–pituitary–adrenal axis, leading to increased glucocorticoid release and subsequent effects on neuronal activity and synaptic plasticity [3].

Experimental and clinical studies have demonstrated that noise induces structural alterations in multiple brain regions. In stress-sensitive areas such as the hippocampus and prefrontal cortex, noise exposure has been associated with reduced neurogenesis, dendritic remodeling, synaptic loss, and alterations in glucocorticoid-mediated signaling [4,5]. In the auditory cortex, noise disrupts tonotopic organization and the balance between excitation and inhibition, reflecting maladaptive plasticity processes that may contribute to the development of conditions such as tinnitus [6,7]. Alterations have also been reported in thalamic regions and in white matter integrity, including changes in myelin and oligodendrocyte populations [8,9]. At the cellular level, these effects involve modifications in dendritic spine density and synaptic protein expression, leading to disruptions in synaptic structure and function [10].

In addition to structural changes, noise exposure induces functional alterations in brain networks. These include impairments in hippocampus-dependent learning and memory, disruptions in hippocampal–prefrontal connectivity, and deficits in executive functions [11,12]. Furthermore, noise has been shown to alter synaptic plasticity mechanisms, such as long-term potentiation and long-term depression, which are essential for information processing and memory consolidation [13].

Multiple interconnected mechanisms, including neuroinflammation, oxidative stress, and glial activation, modulate these structural and functional alterations. Microglial reactivity and astrocytic responses contribute to synaptic remodeling and may exacerbate neuronal dysfunction under chronic noise exposure [14].

Collectively, these changes associated with noise exposure impact cognitive processes, central nervous system structures, and higher-order behavioral functions, including learning, memory, attention, and emotional regulation. Given the increasing prevalence of noise exposure and its broad neurobiological impact, achieving a comprehensive understanding of its effects is essential. Therefore, this review aims to provide an integrated overview of the structural and functional brain changes induced by noise exposure, spanning from cellular and molecular mechanisms to cognitive and behavioral outcomes.

### Search Method

This review used a narrative approach to synthesize current evidence on the neurobiological effects of noise exposure. We conducted a comprehensive literature search across major electronic databases, including PubMed and Web of Science.

Search terms were selected to capture studies addressing both auditory and non-auditory effects of noise exposure on the central nervous system. Keywords included combinations of: “noise exposure,” “brain,” “central nervous system,” “hippocampus,” “prefrontal cortex,” “neuroinflammation,” “synaptic plasticity,” “neurogenesis,” “microglia,” “astrocytes,” “oxidative stress,” “HPA axis,” “cognition,” and “behavior.” Boolean operators (AND, OR) were applied to refine the search and identify relevant publications.

The search included original research articles and reviews published in English, with particular emphasis on studies involving experimental animal models and human data relevant to brain structure, function, and behavior. Priority was given to studies providing mechanistic insights at molecular, cellular, and circuit levels, as well as those examining cognitive and behavioral outcomes associated with noise exposure.

## 2. Structural Brain Changes After Noise Exposure

### 2.1. Hippocampal Volume Reduction

The hippocampal region (HIP) is a bilateral, S-shaped structure located in the medial temporal lobe, comprising the dentate gyrus (DG), the Cornu Ammonis (CA1–CA4) subfields, the subicular complex (including the presubiculum, subiculum, postsubiculum, and parasubiculum), and the entorhinal cortex. The parahippocampal gyrus, although strongly associated with the hippocampal region, is considered a transitional area between the hippocampal formation and the neocortex. These interconnected regions form a complex circuit that integrates inputs from the neocortex, amygdala, striatum, and thalamus, and is functionally associated with the limbic system, including connections with the hypothalamus, especially the mammillary bodies [3,15,16]. The DG is one of the principal neurogenic niches in the adult brain, supporting continuous neurogenesis throughout life [17].

Functionally, the HIP is critically involved in learning and memory processes, emotional regulation, attention, and motivational states [18]. Although it is not part of the classical auditory pathway, the HIP receives both direct and indirect inputs from central auditory structures, allowing acoustic information to modulate its activity [3,17,18,19]. Consequently, chronic noise exposure has been shown to affect hippocampal integrity, particularly within the DG, a region highly sensitive to stress-related stimuli [20].

A growing body of evidence indicates that prolonged exposure to high-intensity noise—95 dB white noise during the day 8 h/d and 75 dB white noise at night 16 h/for 40 days—in male Winstar rats is associated with structural alterations in the hippocampus, including volume reduction. Experimental studies in rodents demonstrate that chronic noise exposure impairs cognitive performance and causes neuronal damage in hippocampal subregions [21]. These structural changes are often paralleled by alterations in central auditory pathways, including neuronal degeneration, disrupted synchrony, and maladaptive axonal sprouting [4]. Importantly, sustained noise exposure has also been linked to long-term suppression of adult hippocampal neurogenesis, a key process underlying structural plasticity and maintenance of volume.

Experimental and translational observations further support these findings. In animal studies with adult male Sprague-Dawley rats unilaterally exposed to narrowband noise (bandwidth 100 Hz) centered at 12 kHz, 126 dB SPL for 2 h have shown that intense noise exposure can impair hippocampal neurogenesis and contribute to cognitive deficits, while human studies have linked chronic noise exposure with adverse cognitive outcomes [5].

Additionally in studies with male rats, sensory deprivation resulting from noise-induced hearing loss, which was induced exposing the left ear of each rat to a 126 dB SPL narrowband noise centered at 12–100 Hz bandwidth—for 2 h, may contribute to hippocampal atrophy by limiting the afferent input necessary for normal synaptic activity and structural maintenance, particularly in processes such as spatial navigation and memory encoding [4].

At the cellular level, noise exposure induces synaptic and network alterations that may precede or contribute to volumetric decline. For instance, studies in animal models have reported disruptions in glutamatergic and GABAergic balance across hippocampal subregions, characterized by increased excitatory and reduced inhibitory synaptic inputs, particularly in CA1, CA3, and DG [22]. These changes promote a state of hyperexcitability and synaptic dysregulation, which can impair synaptic plasticity mechanisms such as long-term potentiation (LTP), especially in the DG [20]. Over time, such alterations may contribute to structural remodeling and volume reduction.

Moreover, noise exposure acts as a potent stressor, activating neuroendocrine responses that directly impact hippocampal structure. The HIP contains a high density of glucocorticoid and mineralocorticoid receptors, making it particularly vulnerable to chronic stress [4,22,23]. Prolonged activation of stress pathways has been associated with dendritic retraction, reduced neurogenesis, and ultimately, hippocampal atrophy. This vulnerability is further exacerbated by early-life stress, which has been shown to accelerate age-related declines in hippocampal plasticity and cellular proliferation [24].

Neuroinflammatory processes also play a critical role in hippocampal volume reduction. Microglial activation, commonly observed following neural injury or chronic stress, has been inversely correlated with hippocampal volume. Increased microglial density reflects ongoing inflammatory responses that may contribute to neuronal loss and impaired structural integrity, thereby promoting neurodegenerative processes [14].

### 2.2. Prefrontal Cortex Structural Remodeling and Thinning

The prefrontal cortex (PFC) plays a central role in top-down regulation of higher-order executive functions. Two major subdivisions within this structure are the medial prefrontal cortex (mPFC) and the orbitofrontal cortex (OFC) [25]. The mPFC comprises heterogeneous neuronal populations with extensive afferent and efferent projections, enabling it to integrate information from multiple brain regions and coordinate adaptive behavioral responses [26]. It is functionally connected to key structures such as the nucleus accumbens, ventral tegmental area, hippocampus, and amygdala through serotonergic, dopaminergic, glutamatergic, and GABAergic pathways [27]. Through these networks, the mPFC contributes to cognitive processes including working memory, decision-making, emotional regulation, and social behavior.

The OFC, in turn, is structurally connected to the contralateral hemisphere via interhemispheric pathways, as well as to association tracts such as the uncinate fasciculus and inferior fronto-occipital fasciculus [28]. Functionally, the OFC is critically involved in reward evaluation, outcome prediction, and behavioral flexibility. It enables updating decisions in response to changing environmental contingencies and plays a key role in inhibiting maladaptive or impulsive response [29].

Disruption of PFC integrity—whether through direct injury or stressors such as chronic noise exposure—leads to impairments in executive function and increases vulnerability to psychiatric and cognitive disorders [25,26,29]. Activation of the hypothalamic–pituitary–adrenal (HPA) axis during stress results in elevated glucocorticoid levels, which exert direct effects on the mPFC, including reduced astrocyte proliferation and survival [30]. These cellular alterations compromise structural support and synaptic homeostasis within prefrontal circuits.

Experimental studies in anesthetized rodents have shown that noise exposure—broadband noise (0.8–20 kHz) was delivered bilaterally for two hours at 115 dB SPL placed 10 cm in front of the anesthetized rat—induces region-specific alterations in cortical organization. While the auditory cortex exhibits increased excitatory activity and synaptic density—changes associated with tinnitus and hyperacusis—the PFC displays reduced functional connectivity and decreased expression of synaptic proteins, suggesting impaired integrative capacity and reduced plasticity [31]. Similarly, in non-human primates, acute noise exposure (105 dB) acts as a stressor that selectively disrupts spatial working memory, a function critically dependent on PFC integrity [12]. These effects have been linked to dysregulated dopaminergic signaling, as excessive dopamine levels in the mPFC impair neuronal firing patterns and disrupt executive processing [32].

In addition to dopaminergic alterations, glucocorticoid-mediated mechanisms contribute to structural remodeling of the PFC. Early-life exposure to noise-induced stress—urban audio files which contain unpredictable noise events with a duration ranging from 18–39 s and spaced by silent intervals ranging from 20–165 s were randomly presented to rats in a 24 h fashion throughout the 15 days post-weaning. The acoustic signal ranging from 70 dB for the background noise to 85–103 dB for the noisy events leads to persistent reductions in astrocyte density within prefrontal regions, with effects that extend into adulthood, indicating long-term structural vulnerability [30]. Chronic exposure to noise, such as traffic-related stressors, has been associated with reductions in cortical volume and thickness, particularly in medial and dorsolateral PFC regions. These morphological changes reflect a loss of neuronal and glial elements, ultimately compromising synaptic plasticity and network efficiency [9].

At the microstructural level, chronic stress induces region-specific dendritic remodeling. In the mPFC, stress reduces apical dendritic length by approximately 20% and decreases dendritic branching, particularly in the anterior cingulate cortex, without significantly affecting basal dendrites [33,34,35]. In contrast, the OFC exhibits an opposing pattern, characterized by increases in apical dendritic length and branching. This divergence suggests that distinct prefrontal subregions respond differentially to stress, with the OFC potentially engaging compensatory or adaptive mechanisms.

Consistent with this notion, noise exposure induces *c-fos* mRNA expression, a marker of neuronal activation, across multiple brain regions. The OFC shows enhanced *c-fos* expression during chronic stress conditions, indicating sustained neuronal activation in response to repeated auditory stimulation [36]. This pattern appears to be independent of direct auditory damage and may reflect region-specific adaptive plasticity mechanisms that distinguish the OFC from other prefrontal and sensory areas.

### 2.3. Auditory Cortex Reorganization and Plasticity

From an anatomical perspective, the auditory cortex (AC) comprises a set of cortical fields that receive major thalamic input from subdivisions of the medial geniculate complex [2]. Rather than representing a single homogeneous structure, the AC consists of multiple specialized regions distributed across the temporal lobe, including primary and secondary auditory areas. These regions are organized according to a core–belt–parabelt architecture, where the primary auditory cortex (A1) is one of several core auditory areas characterized by precise tonotopic organization and strong thalamocortical projections that support hierarchical processing of acoustic information [2,37].

These anatomically distinct fields are integrated into a highly interconnected cortical network, specific thalamocortical and corticocortical connectivity patterns govern information flow. Such connections follow laminar and hierarchical principles, enabling both feedforward and feedback interactions across auditory regions [2]. Consistent with this organization, neuronal activity in the AC is characterized by sparse coding and high temporal precision, whereby acoustic information is represented through coordinated population dynamics rather than uniform neuronal activation. This encoding strategy supports efficient processing of complex auditory stimuli [38].

This highly specialized system is particularly vulnerable to excessive or prolonged acoustic stimulation. At the microcircuit level, exposure to intense noise induces cell type–specific alterations in neuronal excitability within layer V of A1. At this region, neurons comprise at least two subtypes: thick-tufted, subcortically projecting Type A, with prominent h-current, and thin-tufted, callosally projecting Type B neurons, which lack prominent h-current [39]. Type A pyramidal neurons exhibit reduced firing frequency, whereas Type B pyramidal neurons show increased sustained activity. In parallel, Martinotti interneurons display enhanced steady-state firing. These differential changes indicate a disruption of the excitation–inhibition balance and a reconfiguration of cortical output dynamics, providing a cellular basis for subsequent alterations in auditory processing and behavior [40].

At the systems level, converging evidence from animal models demonstrates that noise exposure induces significant reorganization of cortical representations in A1. In rodents, chronic exposure to low-level noise—broad-band “white” noise (pseudo-random, 4–45 kHz in frequency range) was broadcasted continuously 24 h—disrupts the orderly tonotopic organization, leading to fragmented and patchy frequency maps [7]. Additionally, there is an expansion of the cortical representation of exposure frequency, associated with reduced frequency selectivity and impaired auditory discrimination, but for other side, chronic low-level ambient noise exposure in adults rats produced a marked reorganization of the auditory cortical tonotopic map without significantly altering neuronal frequency tuning [6,7,41]. In studies with exposed rats at determinate frequency shown that increased cortical representation does not necessarily enhance functional encoding but may instead reflect maladaptive plasticity [41].

In contrast, exposure to structured acoustic environments—characterized by defined spectral and temporal regularities—can induce adaptive forms of cortical plasticity. Under these conditions, A1 reorganizes in a manner that enhances the representation of behaviorally relevant sounds, suggesting that the AC dynamically adjusts its coding properties according to the statistical structure of the acoustic environment [42].

During critical developmental periods, prolonged exposure to moderate levels of white noise produces long-lasting alterations in auditory cortical function. These include changes in auditory sensitivity, modifications in oscillatory activity across θ, β, and γ frequency bands, and reduced expression of glutamatergic receptor subunits, including AMPA and NMDA receptors [43]. Such alterations indicate disruptions in synaptic excitability and plasticity mechanisms, which may impair the maturation of central auditory circuits even in the absence of overt peripheral damage.

Studies were conducted with male Wistar rats exposed to acoustic trauma which was induced in the left ear by exposing them to a 10 kHz tone with an intensity of 125 dB SPL for 1 h and then was used an acoustic startle stimulus as a white noise—95 dB SPL—before the rats were exposed to four continuous pure tones—each at 60 dB—with frequencies of 4, 8, 16 and 32 Hz. As a result, its tonotopic map was disorganized. Also, they demonstrated that hearing loss significantly reduces the monotonicity of tone-evoked activities. These findings support the notion that auditory cortex reorganization induced by noise exposure reflects a predominantly central process, driven by activity-dependent plasticity mechanisms rather than solely by peripheral auditory injury [6].

### 2.4. Cingulate Cortex and Thalamic Alterations

The cingulate cortex (CC) is a central component of the limbic system, characterized by extensive reciprocal connections with cortical and subcortical structures, including the thalamus (Th), which support its role in integrative neurobiological processes [44]. Functionally and anatomically, the CC is subdivided into distinct regions, including the anterior cingulate cortex (ACC), midcingulate cortex (MCC), posterior cingulate cortex (PCC), and subgenual cingulate cortex (sgACC), each associated with specific connectivity patterns and functional specializations [45].

The thalamus, located in the central region of the brain, is composed of multiple nuclear groups, including anterior, ventral, medial, lateral, midline, and intralaminar nuclei, as well as the medial and lateral geniculate bodies [46]. Several of these nuclei are functionally integrated into limbic circuits through connections with the hypothalamus, amygdala, and cingulate cortex [47], supporting their involvement in emotional and cognitive processing.

Thalamo-cingulate interactions play a critical role in affective regulation, action–outcome learning, and memory integration [48]. Due to their integrative function, these networks are particularly sensitive to stressors such as noise. Both acute and chronic acoustic exposure have been shown to activate thalamocortical pathways, leading to functional reorganization that affects both the CC and Th.

Experimental evidence indicates that noise exposure—122 dB for 2 h—induces significant alterations in the ACC, a region highly involved in emotional regulation and cognitive control. In animal models, acoustic stress is associated with reduced functional connectivity between the ACC and multiple brain regions, including the medial geniculate body (MGB), other cingulate subregions, and broader cortico-subcortical circuits. These connectivity changes are accompanied by network-level reorganization that has been interpreted as a compensatory response; however, it is often associated with behavioral outcomes resembling anxiety- and depression-like phenotypes. Notably, such alterations can persist over time, suggesting incomplete or maladaptive adaptation processes within ACC networks following repeated or intense noise exposure [49,50].

At the thalamic level, the medial geniculate body is particularly vulnerable to acoustic overstimulation. Exposure to high-intensity noise induces functional alterations in the MGB that impair its capacity to process repetitive auditory stimuli, disrupting habituation mechanisms. This results in an imbalance between the detection of salient auditory signals and the suppression of background noise, a dysfunction that has been associated with the development of tinnitus, as well as cognitive and emotional disturbances [51].

In addition to its role in auditory processing, thalamic dysfunction contributes to broader neuroendocrine dysregulation. Altered thalamic activity under conditions of intense or chronic noise exposure has been linked to disruptions in HPA axis regulation. Initially, excessive thalamic activation may lead to a blunted stress response; however, with repeated exposure, this pattern shifts toward maladaptive regulation, characterized by sustained, exaggerated HPA axis activation. This transition reflects impaired adaptation to chronic stress and may contribute to long-term neurobiological and behavioral alterations [52,53,54].

### 2.5. Myelin Integrity Disruption and Oligodendrocyte Dysfunction

Oligodendrocytes (OLCs) originate from oligodendrocyte precursor cells (OPCs), which differentiate and migrate throughout the central nervous system (CNS) to generate mature myelinating cells [55]. Within the CNS, OLCs are the primary glial population responsible for the formation of myelin sheaths, enwrapping axons and producing multiple myelin segments relative to their cellular size. As such, they constitute the exclusive source of myelination in the CNS [56,57].

Myelin is essential for efficient neural communication, as it enables saltatory conduction along axons, thereby increasing the speed and reliability of electrical signal transmission. Beyond its classical role, accumulating evidence indicates that myelin also sports axonal metabolism and modulates neuronal activity, influencing higher-order brain functions [58,59,60,61,62,63].

Stressors, including chronic noise exposure, have been identified as factors that can disrupt OLC function and compromise myelin integrity. Experimental studies demonstrate that stress-related conditions induce region-specific alterations in myelination, particularly in structures such as the corpus callosum and PFC. These alterations include compensatory changes in the expression of myelin-associated genes, suggesting that myelin regulation is dynamically modulated and not uniformly affected across brain regions [8,64].

At the cellular level, stress impairs OPC differentiation into mature OLCs, thereby reducing the availability of cells capable of sustaining and remodeling myelin sheaths. In addition to these effects on cellular populations, microstructural abnormalities have been reported, including disruption of the nodes of Ranvier and decreased oligodendroglial activity. These changes compromise the structural organization of myelinated fibers and impair the efficiency and fidelity of axonal conduction [65]. Overall, these findings indicate that stress-related processes associated with noise disrupt both oligodendrocyte dynamics and myelin integrity, leading to alterations in white matter structure and neural signaling.

### 2.6. Reduced Dendritic Spine Density and Morphological Alterations

Dendritic spines (DSs) are micrometer-scale protrusions of the dendritic membrane that serve as the primary postsynaptic sites of excitatory glutamatergic synapses in the brain [66,67]. These structures form compartmentalized biochemical and electrical microdomains, enriched with postsynaptic densities containing glutamate receptors, predominantly AMPA and NMDA receptors [67,68]. The number, size, and morphology of DSs are highly dynamic and tightly regulated by synaptic activity, reflecting their critical role in synaptic plasticity and information processing [66,68].

Due to their activity-dependent nature, DSs are particularly vulnerable to stressors such as noise exposure and its associated neurobiological consequences. Experimental evidence indicates that exposure to high-intensity noise (e.g., 100 dB for 10 days) induces significant structural alterations in pyramidal neurons of the AC, including a marked reduction in DS density across multiple cortical layers [10].

Similar effects have been observed in non-auditory regions. In models of noise-induced hearing loss combined with stress, reductions in spine density have been reported along apical dendrites of pyramidal neurons in the PFC. These changes are accompanied by alterations in both glutamatergic and GABAergic synaptic markers, indicating disruption of synaptic balance [10,31,35]. In addition to reductions in spine number, morphological shifts have been described, characterized by an increased proportion of small, immature spines and a corresponding decrease in larger, stable spine types. This pattern suggests impaired synaptic maturation and reduced stability of excitatory synapses.

These structural alterations result in a decrease in functional excitatory synaptic contacts and contribute to an imbalance between excitatory and inhibitory neurotransmission. Such disruptions impair synaptic plasticity mechanisms and compromise the efficiency of neuronal communication, particularly in circuits involved in higher-order cognitive processing.

### 2.7. Dendritic Atrophy in Hippocampus and PFC

Exposure of the HIP and PFC to noise is associated with significant alterations in synaptic plasticity, reflected by a reduction in LTP, a process closely linked to dendritic integrity and synaptic connectivity [11,13,69,70]. These impairments are accompanied by biochemical alterations in signaling pathways that regulate activity-dependent structural remodeling. At the morphological level, noise exposure induces pronounced dendritic atrophy in pyramidal neurons, predominantly affecting apical dendritic compartments. This includes dendritic retraction, reduced dendritic length, and decreased branching complexity, ultimately compromising synaptic integration. Converging evidence indicates that dendritic atrophy in these regions is consistently accompanied by reduced dendritic spine density, further decreasing excitatory synaptic contacts [13,31,70,71]. The combined effect of dendritic retraction and spine loss results in diminished synaptic efficacy and impaired network communication. These structural alterations disrupt neuronal circuitry within the HIP and PFC, contributing to deficits in memory, learning, executive function, and emotional regulation.

### 2.8. Alterations in Synaptic Protein Expression (PSD-95, Synaptophysin, NMDA and AMPA Receptors)

Chronic noise exposure induces significant alterations in the expression of key synaptic proteins involved in excitatory neurotransmission. In the HIP, reduced expression of the NR2B subunit of NMDA receptors has been consistently reported across multiple subregions. This reduction is associated with increased Tau hyperphosphorylation and elevated neuronal apoptosis, suggesting a link between impaired glutamatergic signaling and neurodegenerative processes [72,73]. Similar decreases in NR2B expression have been observed in the PFC, along with reduced NR2A levels in the contralateral inferior colliculus, indicating widespread disruption of NMDA receptor-mediated synaptic function [31,74]. Imbalances in glutamatergic and GABAergic neurotransmission further accompany these alterations, reinforcing the presence of synaptic dysfunction [73].

Alterations in AMPA receptor subunits have also been described. In moderate-level noise exposed at 65 dB SPL rodents, decreased levels of GluR1 and its phosphorylated form (p-GluR1) have been observed in the HIP, reflecting impaired postsynaptic signaling and reduced synaptic efficacy [11]. Notably, synaptic vulnerability appears to depend on receptor composition. Synapses enriched in calcium-permeable AMPA receptors (CP-AMPARs) exhibit greater susceptibility to structural and functional damage induced by noise. In contrast, synapses with higher GluA2 content show relative resistance, highlighting the role of synaptic microarchitecture in determining the impact of acoustic stress [75].

Changes can occur in structural synaptic proteins such as postsynaptic density protein (PSD) complex which is critical for synaptic strength and plasticity in excitatory neurons. Here, the scaffolding postsynaptic density protein 95 (PSD-95) plays a crucial role as It organizes key PSD components essential for synaptic signaling, development, and survival [76,77]. PSD-95 and synaptophysin, further support the presence of noise-induced synaptic remodeling. In developmental models, prenatal exposure to noise produces divergent effects depending on its temporal structure. Arrhythmic noise exposure reduces PSD-95 and synaptophysin expression in the HIP, consistent with decreased synaptic density and efficiency. In contrast, rhythmic noise exposure increases the expression of these proteins, suggesting enhanced synaptic organization and potentially adaptive plasticity mechanisms [78,79].

In other brain regions, such as the AC, noise-induced hearing loss has been associated with increased expression of PSD-95 and synaptophysin, which may reflect compensatory synaptic responses to altered sensory input [31]. These region-specific differences indicate that synaptic protein regulation following noise exposure is not uniform but instead reflects a balance between maladaptive and compensatory plasticity mechanisms across distinct neural circuits.

### 2.9. Dysregulated Microglial Synaptic Pruning

Microglia (MG) constitute the primary resident immune cell population of the CNS and are widely distributed throughout the brain. They maintain close interactions with neuronal elements through highly ramified processes, enabling continuous surveillance of the neural microenvironment. Under physiological conditions, MG predominantly exhibit a ramified morphology associated with homeostatic functions. However, in response to stress, injury, or infection, MG undergo a phenotypic shift toward an activated, amoeboid state characterized by increased motility, proliferation, and phagocytic activity [80,81].

MG are particularly sensitive to noise, which can induce neuroinflammatory responses that directly impact synaptic function and plasticity [82]. In animal models exposed to chronic noise—adult male were exposed to 100 dB white noise 4 h/day for 30 days—activation of the NLRP3 inflammasome has been reported in the HIP, accompanied by elevated levels of pro-inflammatory cytokines [83]. Sustained microglial activation has also been observed across auditory regions and hippocampal subfields, with differential effects reported between DG and CA3 compared to CA1, suggesting region-specific vulnerability [14].

Activated MG modulates synaptic connectivity by releasing inflammatory mediators and directly interacting with synaptic elements, influencing synaptic stability, efficacy, and number. Importantly, these alterations occur in the absence of widespread neuronal loss, indicating that noise exposure primarily induces pathological synaptic remodeling rather than overt neurodegeneration [84,85,86].

At the molecular level, microglia-mediated synaptic pruning is tightly regulated by receptor-mediated signaling pathways. Disruption of these mechanisms contributes to aberrant synaptic elimination. For instance, deletion of signal regulatory protein alpha (SIRPα), a receptor that recognizes “self” signals to protect synapses from phagocytosis, results in significant reductions in synaptic density in cortical and hippocampal regions, particularly in CA1, without affecting neuronal survival. These findings highlight the role of microglial signaling pathways in maintaining synaptic integrity [87].

Additional regulatory pathways include purinergic signaling mediated by P2Y12 receptors, which are essential for microglial surveillance and synaptic modulation. Impairment of P2Y12 function, as observed under conditions of injury or chronic stress, is associated with increased microglia-mediated synaptic elimination, leading to excessive or inappropriate pruning [88].

Functionally, these alterations can disrupt the balance between excitatory and inhibitory synapses. In mice models of chronic noise-induced stress—80 dB and a frequency range between 10 and 10,000 Hz for 2 h per day for six consecutive weeks—or anxiety—exposed to chronic moderate noise to 85 dB for 4 h per day for 28 days—enhanced microglial phagocytosis of inhibitory synapses has been reported in the lateral amygdala, resulting in a shift toward hyperexcitability. This imbalance contributes to the emergence of anxiety- and depression-like behaviors, linking microglial dysfunction with behavioral outcomes following chronic noise exposure [82,89].

### 2.10. Impaired Hippocampal Neurogenesis: Reduced Proliferation, Differentiation, and Maturation

Chronic noise—for 15 days—exposure has been consistently associated with impairments in HIP-dependent memory processes. Stressors during critical periods of brain development exert long-lasting effects on hippocampal structure, including reductions in neuronal number, dendritic arborization, and increased neuronal cell death, particularly within the DG [90,91].

Experimental studies demonstrate that both noise exposure and noise-induced hearing loss produce persistent alterations in adult neurogenesis, especially within the subgranular zone of the DG. At the cellular level, these effects are characterized by a significant reduction in cell proliferation, as evidenced by decreased expression of proliferation markers such as Ki67 and BrdU. Ki67 is a nuclear protein expressed during the active phases of the cell cycle and serves as a marker of ongoing cell proliferation, whereas BrdU is a thymidine analog that incorporates into DNS of diving cells during the S phase, enabling the identification of newly generated cells. Reduced Ki67 and BrdU labeling following noise exposure suggests impaired progenitor cell proliferation and diminished adult hippocampal neurogenesis. Notably, this reduction persists for extended periods following exposure, indicating a sustained disruption rather than a transient response. In parallel, elevated corticosterone levels have been observed, reflecting activation of the HPA axis and suggesting that neuroendocrine stress mechanisms contribute to the suppression of neurogenic processes [3,5,14,92,93,94,95].

In addition to reduced proliferation, noise exposure impairs neuronal differentiation. A decrease in the number of doublecortin-positive (DCX^+^) immature neurons has been reported, indicating that fewer neural precursor cells progress toward neuronal lineage commitment. As a result, the integration of newly generated neurons into DG circuits is markedly diminished. Similar to the effects on proliferation, these alterations are long-lasting and contribute to a sustained reduction in adult neurogenesis [5,14,95].

Alterations in neuronal maturation further exacerbate these effects. Newly generated neurons exhibit simplified morphological features, including thinner dendritic processes and reduced branching complexity, indicative of impaired synaptic maturation and integration. These changes are not uniform across neuronal populations; younger neurons may display partial compensatory responses, whereas more mature neurons tend to exhibit more pronounced structural deterioration. Both patterns ultimately compromise network functionality and negatively impact memory processes [3,14,96].

Importantly, evidence suggests that noise-induced hearing loss accelerates the decline in hippocampal neurogenic capacity more rapidly than aging alone. This finding underscores the role of chronic noise exposure, in combination with stress and neuroinflammatory processes, as a potent driver of hippocampal dysfunction and reduced regenerative potential [81].

### 2.11. Microglial Activation and Neuroinflammatory Responses

MC constitutes the resident macrophage population of the CNS and are distinguished from other glial cells, such as astrocytes (ATs) and OLCs, by their developmental origin, morphology, gene expression profiles, and functional roles [80,97,98]. These cells play a central role in the brain’s response to injury, pathological conditions, and intense stimuli, including chronic or high-intensity noise exposure. In response to such stressors, microglia undergo a phenotypic transition from a ramified, surveillance state to an activated amoeboid morphology, characterized by increased motility, migration toward affected regions, and the release of a broad range of neuroactive mediators with either neurotoxic or neuroprotective effects [99].

The activated amoeboid phenotype exhibits enhanced phagocytic capacity and is typically induced by pro-inflammatory molecules such as lipopolysaccharide (LPS) which induces the production of various pro-inflammatory cytokines such as interferon-gamma (IFN-γ) and other substances such as amyloid-beta (Aβ) which is a peptide generated by the proteolytic processing of the amyloid precursor protein (APP) and in chronic noise exposure, has been observed an increase in the levels of the Aβ1-40 and Aβ1-42 fragments, along with an increase in the expression of the β-site amyloid precursor protein cleavage enzyme 1 (BACE1); and α-synuclein [100,101,102]. In the context of noise exposure, this activation is associated with a robust neuroinflammatory response in the HIP, including elevated levels of pro-inflammatory cytokines such as group of interleukins (IL) like IL-1β, IL-6, IL-18, and Tumor Necrosis Factor-alpha (TNF-α) among them, this cytokine has been shown to play a central role in organizing the inflammatory response in the brain [103]. Activated microglia also produces reactive oxygen species (ROS) and upregulates major histocompatibility complex class II molecules, contributing to antigen presentation and amplification of the inflammatory cascade [83,85,104,105].

Microglial activation is commonly assessed using molecular markers such as Iba-1, an ionized calcium-binding adaptor protein indicative of microglial reactivity, and cluster of differentiation 68 (CD68), a transmembrane glycoprotein expressed by human monocytes and tissue macrophages that indicate phagocytic activity [106], which is associated with pro-inflammatory phenotypes. In animal models exposed to noise—e.g., broadband noise at 123 dB SPL for 2 h—increased expression of these markers has been consistently observed across hippocampal subregions, including DG, CA1, and CA3 [14,85,89,107].

Morphologically, activated microglia in these regions exhibit shortened, retracted processes, enlarged cell bodies, and increased phagocytic activity. The changes indicate a shift toward a reactive state that alters neuron–glia interactions and contributes to local circuit dysfunction. Functionally, such activation is associated with increased synaptic remodeling, including enhanced phagocytosis of synaptic elements, which can disrupt the excitation–inhibition balance and impair neural network stability [14,85,89,107].

### 2.12. Astrocytic Activation and Functional Dysregulation

AT’s represent the most abundant glial population in the CNS and exhibit highly ramified morphologies that enable extensive interactions with neurons, synapses, and the vasculature. They play essential roles in maintaining brain homeostasis by providing metabolic and trophic support, regulating synapse formation and function, participating in synaptic pruning, and contributing to neuroimmune responses. Under pathological conditions—including neurodegeneration, trauma, and chronic stress—ATs undergo a process known as reactive astrogliosis, characterized by pronounced morphological and molecular changes. Hallmarks of this state include cellular hypertrophy and upregulation of glial fibrillary acidic protein (GFAP) [108,109,110].

In the context of noise exposure, astrocytic hypertrophy has been observed in hippocampal regions such as the DG, CA3, and CA1, where astrocytes exhibit elongate, more complex processes. This increased arborization reflects a form of structural plasticity, likely representing an adaptive response aimed at maintaining synaptic surveillance and regulating neuron–glia–vascular interactions under sustained stress conditions [111].

Reactive astrocytes can be broadly categorized into two functional phenotypes: A1 and A2. A1 astrocytes exhibit pro-inflammatory and neurotoxic properties, whereas A2 astrocytes are associated with anti-inflammatory and neuroprotective functions. High-intensity noise exposure promotes microglial activation, which in turn induces a shift toward the A1 astrocytic phenotype within the HIP, without a corresponding increase in A2 astrocytes [109,112,113]. This imbalance favors a pro-inflammatory environment that contributes to neural dysfunction.

At the molecular level, noise exposure activates signaling pathways such as nuclear factor-kappa B (NF-κB) which is a transcription factor that regulates genes associated with inflammation mediated by immune cells [114]; and the HPA axis, leading to increased levels of pro-inflammatory cytokines, including IL-18 and TNF-α, and upregulation of astroglial markers such as complement component C3 and GFAP [112,115]. These changes reflect sustained astrocytic reactivity that can persist for extended periods, promoting chronic neuroinflammation and contributing to dysfunction in circuits associated with anxiety and emotional regulation [85].

Astrocytes also play a critical role in glutamate homeostasis through the expression of high-affinity transporters, including glutamate/aspartate transporter (GLAST) and glutamate transporter 1 (GLT1) (EAAT1 and EAAT2 in humans), which are essential for clearing extracellular glutamate and preventing excitotoxicity. Under conditions of chronic stress and noise exposure—e.g., 4 kHz continuous pure tone at 105 dB sound pressure level (SPL) for 30 min—the expression of genes encoding these transporters (e.g., SLC1A2 and SLC1A3) is reduced, impairing glutamate uptake. This dysfunction leads to extracellular glutamate accumulation, increased excitotoxic stress, neuronal damage, and further amplification of the pro-inflammatory environment [116,117,118].

## 3. Functional Brain Changes After Noise Exposure

### 3.1. Reduced Hippocampal–PFC Connectivity

The structural alterations described above are accompanied by a wide range of functional disturbances that affect neuronal communication, neuroendocrine regulation, metabolic homeostasis, and cognitive processing. These functional changes emerge from the interaction of multiple molecular and cellular mechanisms and collectively contribute to the adverse neurological and behavioral outcomes associated with chronic noise exposure.

Chronic noise acts as a persistent stressor that promotes sustained activation of the HPA axis. This process involves sequential release of CRH, ACTH, and corticosterone, leading to prolonged exposure of neural circuits to stress hormones. Notably, these endocrine responses appear to be robust, occurring independently of sex and circadian variation [119]. Sustained glucocorticoid signaling has been shown to disrupt synaptic integrity and network-level communication between the HIP and PFC.

At the molecular and cellular level, noise-induced stress is associated with tau hyperphosphorylation in the HIP, a process linked to synaptic dysfunction and impaired neuronal transmission. These alterations contribute to reduced functional coupling between the HIP and mPFC, reflected in decreased coherence and synchronization of neuronal activity under chronic stress [120,121]. Such reductions in functional connectivity indicate impaired coordination between these regions, which is essential for cognitive processes such as working memory and executive control.

Additional factors, including noise-induced hearing loss, further exacerbate these connectivity deficits. Alterations in hippocampal neurogenesis and disruptions in place cell firing patterns impair the integration of spatial and contextual information, weakening coordinated activity within hippocampal–prefrontal circuits [3,122]. These changes are not restricted to this circuit but extend to broader limbic networks, indicating widespread disruption of circuit-level organization.

### 3.2. Impaired Executive Control Networks

Large-scale brain networks coordinate neural activity during both resting and task-related states, supporting cognitive functions and internal mentation. Among these, executive control networks—centered in the PFC—interact dynamically with other intrinsic systems such as the Default Mode Network (DMN), which is involved in self-referential processing and memory-related functions. Proper cognitive performance depends on the balanced interaction between these networks, particularly the ability of executive regions to regulate DMN activity.

Under conditions of chronic stress and neuroinflammation, such as those induced by prolonged noise exposure, this balance becomes disrupted. Alterations in DMN connectivity have been consistently reported, particularly involving prefrontal regions and their connections with limbic structures, including the HIP [123,124,125]. These changes reflect a loss of efficient network segregation and integration, impairing the capacity of executive control systems to modulate internally driven processes.

Functionally, this dysregulation is associated with reduced top-down control, leading to deficits in attention, working memory, and cognitive flexibility. At the network level, impaired coordination between executive regions and the DMN results in persistent activation of internally oriented processing, which interferes with goal-directed behavior.

These connectivity alterations can manifest in both short- and long-term contexts, indicating that executive control networks are highly sensitive to sustained stress and neuroinflammatory states.

### 3.3. Reduced LTP and Altered LTD

Glutamatergic neurotransmission, particularly mediated by NMDA receptors, plays a central role in synaptic plasticity mechanisms underlying memory, cognition, sensory processing, and the formation of neuronal networks during brain development [126,127]. Additional glutamatergic receptors, including AMPA, kainate (KAR), and metabotropic glutamate receptors (mGluRs), also contribute to these processes by regulating long-term synaptic plasticity [128].

LTP and LTD represent complementary forms of long-term synaptic modification that are essential for learning and memory. Both processes depend on intracellular Ca^2+^ dynamics and can occur at the same synapse, with their balance determining synaptic strength and network adaptability [128,129,130,131].

Under conditions of stress, such as chronic noise exposure, synaptic function becomes compromised, leading to deficits in hippocampus-dependent learning and memory. These impairments are closely associated with a reduction in LTP induction, accompanied by downregulation of key signaling molecules involved in synaptic potentiation [11]. This reduction in synaptic strengthening limits circuits’ ability to encode and retain information.

In parallel, stress exposure facilitates LTD. In animal models, noise, and early-life stress—including prenatal exposure—have been shown to enhance NMDA- and mGluR-dependent LTD, particularly in the CA1 region of the dorsal HIP, while simultaneously impairing LTP induction [11,127,132,133]. This shift toward synaptic weakening disrupts the balance of plasticity mechanisms.

Functionally, the predominance of LTD over LTP interferes with synaptic stability and information storage, impairing hippocampus-dependent memory retrieval and contributing to cognitive deficits associated with chronic stress conditions.

### 3.4. Altered Neural Oscillations (Theta, Gamma, Alpha)

Other Alterations in auditory processing induced by noise exposure can be assessed using electrophysiological biomarkers such as the Auditory Brainstem Response (ABR), which reflects neural conduction along the auditory pathway [134]. In animal models, reductions in wave I amplitude—representing synaptic transmission between inner hair cells and auditory nerve fibers—have been reported, indicating cochlear synaptopathy. In humans, this parameter is less consistent; instead, increased latency of wave V has been observed, suggesting impairments in temporal processing and neural conduction velocity within the brainstem. These changes have been associated with compensatory central mechanisms linked to auditory hypersensitivity and tinnitus [134,135,136].

At the cortical level, neural activity is organized through oscillations across multiple frequency bands, particularly theta, alpha, and gamma. The interaction between these oscillations supports cognitive functions such as memory, attention, and executive control. Theta oscillations are involved in large-scale temporal coordination, alpha rhythms regulate cortical excitability, and gamma oscillations support local synaptic processing and information integration [137].

Noise exposure alters these oscillatory dynamics, affecting both power and coherence across frequency bands. Changes in theta activity and reductions in gamma coherence have been reported, indicating disrupted coordination between local and long-range neural activity [138].

In clinical and experimental models, tinnitus has been associated with increased gamma-band activity in the AC, reflecting abnormal cortical excitability and persistent auditory perception. This activity has also been linked to enhanced coupling between auditory and limbic regions [139,140].

Alterations in cross-frequency coupling, particularly theta–gamma interactions between the HIP and PFC, have been reported under conditions of stress and depressive-like phenotypes. These changes are associated with impairments in memory encoding, synaptic plasticity, and interregional communication [141].

### 3.5. Neurotrophic Dysregulation

Neurotrophic signaling is essential for maintaining synaptic plasticity, neuronal survival, and circuit stability. Among neurotrophic factors, BDNF plays a central role in dendritic growth, spine formation, synaptic stabilization, and modulation of LTP. Its expression is tightly regulated by intracellular pathways, including cyclic AMP response element-binding protein (CREB)-dependent transcription, and is highly sensitive to stress-related signaling.

Chronic stress reduces BDNF expression in the HIP and PFC through sustained glucocorticoid exposure and disruption of CREB-mediated transcriptional regulation. These changes are associated with dendritic atrophy, reduced spine density, and impaired neurogenesis, indicating a direct link between neurotrophic signaling and structural plasticity [142].

In noise exposure—e.g., 85 dB—similar alterations in BDNF signaling have been reported. Noise-induced stress activates the HPA axis and promotes conditions that suppress BDNF expression, contributing to deficits in synaptic maintenance and neuronal integrity. These effects are further compounded by neuroinflammatory and oxidative processes. Chronic noise exposure increases ROS production and elevates pro-inflammatory cytokines, which interfere with BDNF signaling pathways and reduces its availability [1,102].

At the synaptic level, disruption of BDNF signaling is associated with alterations in glutamatergic transmission and plasticity-related mechanisms, including impaired LTP. These changes affect synaptic efficacy and weakening communication within neural circuits. The combined influence of glucocorticoid overexposure, inflammation, and oxidative stress creates a molecular environment that limits neuronal resilience and plasticity.

### 3.6. Altered Neurotransmitter Release and Synaptic Signaling

Noise exposure alters synaptic function by disrupting neurotransmitter dynamics, particularly affecting the balance between excitatory and inhibitory signaling. This imbalance represents a key mechanism through which stressors translate molecular alterations into circuit dysfunction.

Chronic noise exposure has been associated with impairments in hippocampus-dependent learning and memory, accompanied by disruptions in synaptic plasticity mechanisms, including reduced LTP. These effects reflect alterations in neurotransmitter release and synaptic signaling that compromise the functional integrity of neural circuits involved in information processing [22].

At the neurochemical level, noise exposure induces region-specific changes in glutamatergic and GABAergic systems. Experimental studies have reported alterations in glutamate and GABA concentrations, as well as in the activity of enzymes responsible for their synthesis and degradation. These changes indicate that noise not only affects neurotransmitter availability but also disrupts their metabolic regulation, leading to impaired synaptic transmission and altered excitation–inhibition balance [143].

In addition to amino acid neurotransmitters, monoaminergic systems are also affected. In rats models noise-induced stress—white noise—110 dB (1000 Hz) for 20 min—produces changes in dopaminergic and serotonergic signaling, as demonstrated by in vivo microdialysis studies showing rapid fluctuations in monoamine levels across brain regions. These alterations influence stress responsiveness, emotional regulation, and behavioral adaptation, reflecting broader disruption of neuromodulatory control over neural circuits [144]. Structural changes previously described, including dendritic remodeling and reduced synaptic connectivity, occur in parallel with these neurochemical alterations, further modifying synaptic efficacy and signal integration within affected regions [145].

### 3.7. HPA/SAM Axes Activation

Noise exposure engages systemic stress-response pathways, particularly the HPA axis and the sympathetic–adrenal–medullary (SAM) system. As a stressor, chronic noise activates hypothalamic nuclei that regulate CRH release, followed by ACTH secretion and subsequent glucocorticoid production. In parallel, activation of the SAM system promotes the release of catecholamines, including adrenaline and noradrenaline, mediating rapid autonomic responses.

Sustained activation of these systems results in prolonged exposure to stress mediators, leading to dysregulation of neuroendocrine signaling and increased allostatic load. Experimental evidence in rats demonstrates that acute noise exposure—30 min at intensities of 80, 85, 90, 95, 100, 105 and 110 dBA and immediately after noise the rats were sacrificed- elevates circulating glucocorticoids and catecholamines, indicating activation of both endocrine and autonomic pathways. In animal models, increased corticosterone levels and recruitment of stress-responsive brain regions further support activation of the HPA axis under noise conditions [146].

Human studies also report associations between noise exposure and altered neuroendocrine biomarkers, reflecting persistent physiological stress responses. These findings indicate that noise-induced activation of stress axes is not limited to acute responses but extends to chronic dysregulation.

In addition to neuroendocrine effects, activation of stress pathways occurs in parallel with inflammatory and oxidative processes. Chronic noise exposure has been associated with increased production of pro-inflammatory mediators, suggesting an interaction between the neuroendocrine and immune systems. This crosstalk contributes to systemic and neural alterations observed under sustained stress conditions [147,148].

### 3.8. Brain Metabolism Alterations

Chronic noise exposure has been associated with disruptions in brain metabolic homeostasis, largely driven by sustained activation of stress-response pathways. Prolonged glucocorticoid exposure alters cellular energy balance, mitochondrial function, and redox status, particularly in metabolically demanding regions such as the HIP and PFC. These alterations impair neuronal bioenergetics, increase oxidative stress, and disrupt metabolic signaling, affecting synaptic function and neuronal performance.

At the molecular level, noise exposure alters key metabolic pathways, including lipid metabolism and inflammation-related processes. Multi-omics analyses have shown that acute high-intensity noise induces significant changes in arachidonic acid metabolism, a pathway closely associated with neuroinflammatory signaling and cellular stress responses. These alterations are accompanied by cognitive deficits and widespread molecular dysregulation, indicating that noise exposure can reprogram brain metabolic processes [149].

Beyond localized effects, noise exposure also impacts systemic and brain-wide metabolic regulation. Experimental studies demonstrate disruptions in energy homeostasis, including alterations in lipid and glucose metabolism pathways. These findings indicate that noise affects not only neuronal metabolism but also broader metabolic networks that support brain function [150].

These metabolic alterations occur in parallel with oxidative and inflammatory processes. Increased production of reactive oxygen species and activation of inflammatory pathways further compromise metabolic stability, reinforcing neuronal vulnerability. The interaction between metabolic dysregulation, stress signaling, and inflammation contributes to impaired cellular function and reduced resilience of neural circuits [151,152,153].

### 3.9. Cognitive and Behavioral Changes

**Figure 1:**
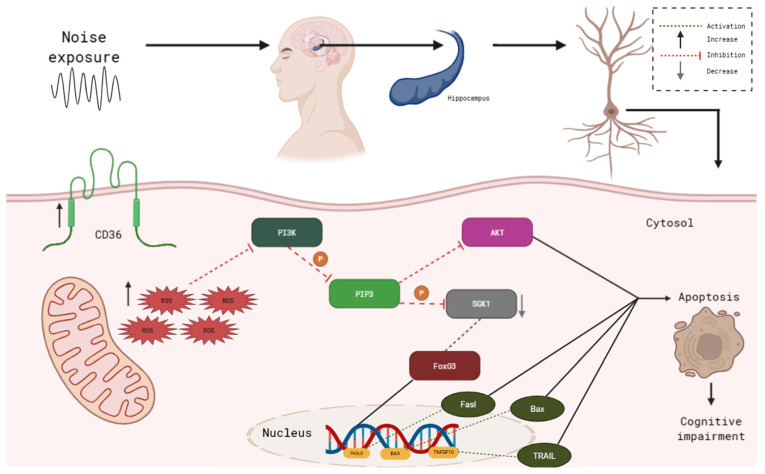
In the neuronal soma, noise exposure is associated with dysregulation of SGK1 and inhibited the PI3K–PIP3–*FoxO3* signaling pathway, resulting in decreased levels of PI3K and its downstream lipid messenger PIP3. Reduced PIP3 availability leads to diminished activation of SGK1, which normally suppresses the transcription factor *FoxO3*. Insufficient SGK1 activity allows *FoxO3* to remain active and translocate to the nucleus, where it promotes transcription of pro-apoptotic genes, including Bax, FasL and TRAIL. In parallel, noise exposure increased CD36 expression which contributes to mitochondrial dysfunction and elevated ROS production. An excess of ROS suppresses the expression levels of the PI3K/AKT pathway leading to apoptosis and finally to cognitive impairment.

The HIP is among the most vulnerable brain regions to noise exposure (Figure 1). Through its interaction with auditory processing and stress-related signaling, chronic noise disrupts adult neurogenesis, particularly in the DG [5]. Experimental studies have consistently shown that noise exposure induces persistent deficits in spatial learning and memory, which can remain detectable months after the cessation of the stimulus. These impairments are associated with reductions in DCX^+^ and Ki67^+^ cell populations, reflecting decreased neuronal proliferation and differentiation, ultimately compromising synaptic plasticity and information encoding [5,11,95].

At the molecular level, noise exposure induces alterations in intracellular signaling pathways that regulate neuronal survival, synaptic integrity, oxide resistance and cell apoptosis such as PI3K/SGK1/*FoxO3* (Phosphatidylinositol 3-kinasa/Serum/Glucorticoid-regulated Kinase 1/*Forkhead box O3*) and PI3K/AKT (Phosphatidylinositol 3-kinasa/Proteín kinase B) (Figure 1). Disruption of these pathways contributes to increased neuronal vulnerability, favoring oxidative stress and apoptotic processes, which together impair hippocampal function and cognitive performance [21,154,155].

**Figure 2:**
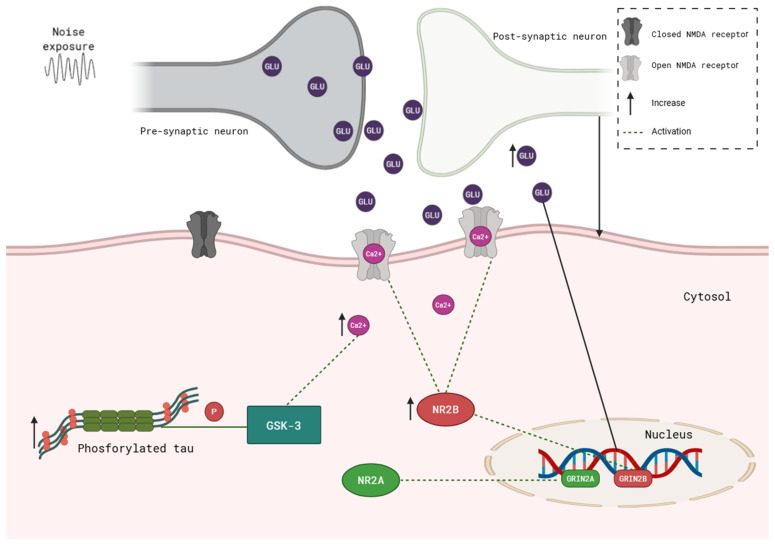
Noise-induced glutamatergic excitatory and tau pathology in hippocampal neurons. Noise exposure increases extracellular glutamate levels, leading to overexpression of the glutamate ionotropic receptor NMDA type subunit 2B gene (*GRIN2B*), which encodes the NR2B subunit of the N-methyl-d-aspartate (NMDA) receptor. This promotes sustained receptor activation and increased Ca^2+^ influx into postsynaptic neurons. Elevated intracellular Ca^2+^ activates downstream kinases such as glycogen synthase kinase-3 beta (GSK-3β), promoting tau hyperphosphorylation (hyperphosphorylated tau), microtubule destabilization, and synaptic dysfunction, ultimately contributing to cognitive impairment. The NR2A subunit, encoded by the *GRIN2A* gene, is also shown because alterations in the balance between NR2A- and NR2B-containing NMDA receptors influence synaptic plasticity and neuronal survival.

Additionally, noise exposure promotes glutamatergic dysregulation and excitotoxic signaling (Figure 2). Increased extracellular glutamate and enhanced activation of N-metil-d-aspartate (NMDA) receptors, particularly those containing the NR2B and NR2A subunits produced by *GRIN2A* and *GRIN2B* genes. These subunits heavily contribute to memory function but NR2B specially contributes to working memory, an overexpression of NR2B can lead to excessive Ca^2+^ influx and activation of downstream kinases. These processes are associated with tau hyperphosphorylation and synaptic dysfunction, which have been linked to impairments in memory performance [156].

Noise exposure activates stress-related systems; this neuroendocrine response is accompanied by increased production of pro-inflammatory cytokines such as IL-6, TNF-α, and IL-1β, contributing to a sustained neuroinflammatory environment. Together, these mechanisms disrupt neural circuits involved in memory and emotional regulation, promoting anxiety- and depression-like phenotypes [157,158,159].

Beyond memory-related processes, noise exposure affects attention, inhibitory control, and executive function. Experimental evidence indicates an imbalance between excitatory and inhibitory neurotransmission, characterized by enhanced glutamatergic activity and reduced GABAergic signaling, which is associated with deficits in attention and cognitive control [160]. In humans, exposure to noise impairs performance in tasks requiring inhibitory control, such as the Stroop test, leading to increased error rates. Furthermore, chronic noise exposure has been associated with reduced reading performance and impaired speech comprehension in children [161,162,163].

At the systems level, noise acts as a cognitive stressor that increases processing demands in the PFC, reducing efficiency in executive function and information processing. This results in slower response times, increased error rates, and decreased performance even in relatively simple cognitive tasks [161,164,165].

## 4. Limitations and Future Perspectives

Despite the growing body of evidence supporting the impact of noise exposure on brain structure and function, several limitations must be acknowledged. First, there is a notable heterogeneity in experimental models, particularly regarding the use of animal versus human studies. While animal models provide critical mechanistic insights at molecular and cellular levels, their translational validity remains limited due to differences in auditory processing, lifespan, and environmental complexity. Conversely, human studies often rely on observational or epidemiological designs, which restrict causal inference and mechanistic interpretation.

A major limitation concerns the variability in noise exposure parameters across studies. Differences in intensity (dB levels), duration (acute vs. chronic), frequency composition, and temporal patterns (continuous vs. intermittent noise) make direct comparisons difficult and may account for inconsistencies in reported outcomes. This variability also complicates the establishment of standardized exposure thresholds relevant to public health.

Additionally, there is a relative scarcity of longitudinal studies evaluating the long-term effects of noise exposure on brain structure and cognitive function. Most available evidence is derived from cross-sectional or short-term experimental designs, limiting the understanding of temporal dynamics, reversibility, and cumulative effects of noise-induced neurobiological alterations. This gap is particularly relevant given that some cognitive and structural impairments may persist months or even years after exposure.

Another important limitation is the insufficient integration of multimodal approaches. Although structural, molecular, and behavioral alterations have been described, few studies simultaneously assess neuroimaging, electrophysiological, biochemical, and behavioral outcomes within the same experimental framework. This limits the ability to establish comprehensive models linking cellular mechanisms to system-level dysfunction and behavioral phenotypes.

Furthermore, potential confounding factors are often not fully controlled, particularly in human studies. Variables such as socioeconomic status, co-exposure to air pollution, baseline stress levels, sleep quality, and individual susceptibility may influence the observed effects of noise on the brain, introducing bias and variability in the results.

Future research should aim to address these limitations through the development of standardized noise exposure protocols, allowing for better reproducibility and comparability across studies. Longitudinal designs are essential to determine the progression and potential reversibility of noise-induced brain alterations. In addition, translational approaches integrating animal models with human neuroimaging and cognitive assessments will be critical to bridge mechanistic findings with clinical relevance.

Emerging methodologies such as multi-omics, advanced neuroimaging techniques, and machine learning-based predictive models represent promising avenues to better characterize the complex interactions between noise exposure, neuroinflammation, metabolic dysregulation, and cognitive impairment. Finally, future studies should explore potential therapeutic and preventive strategies, including pharmacological interventions targeting oxidative stress and neuroinflammation, as well as environmental and behavioral modifications aimed at reducing noise exposure and enhancing resilience to stress.

## 5. Conclusions

Noise exposure acts as a significant stressor capable of inducing widespread neurobiological alterations that extend beyond the auditory system, affecting key regions such as the HIP and PFC involved in cognition, behavior, and emotional regulation. The evidence supports a multifactorial framework in which neuroendocrine activation, neuroinflammation, oxidative stress, and glial dysfunction interact to disrupt synaptic integrity, neural connectivity, and circuit function.

Recognition of noise exposure as a determinant of brain health underscores the need for preventive and mitigation strategies to reduce its neurobiological and behavioral impact.

## Data Availability

All research data is in the main manuscript.

## Abbreviations

A1	Primary auditory cortex
AC	Auditory cortex
ACC	Anterior cingulate cortex
ACTH	Adrenocorticotropic hormone
ATs	Astrocytes
BDNF	Brain-derived neurotrophic factor
CA	Cornu Ammonis
CC	Cingulate cortex
CNS	Central nervous system
CRH	Corticotropin-releasing hormone
DG	Dentate gyrus
DMN	Default mode network
DSs	Dendritic spines
GFAP	Glial fibrillary acidic protein
HIP	Hippocampal region
HPA	Hypothalamic–pituitary–adrenal
LTD	Long-term depression
LTP	Long-term potentiation
MCC	Midcingulate cingulate cortex
MG	Microglia
MGB	Medial geniculate body
ML	Myelin
mPFC	Medial prefrontal cortex
OFC	Orbitofrontal cortex
OLCs	Oligodendrocytes
OPC	Oligodendrocyte precursor cell
PCC	Posterior cingulate cortex
PFC	Prefrontal cortex
ROS	Reactive oxygen species
SAM	Sympathetic-adrenal-medullary
sgACC	Subgenual cingulate cortex
Th	Thalamus
